# Intra-observer reliability for measuring first and second toe and metatarsal protrusion distance using palpation-based tests: a test-retest study

**DOI:** 10.1186/s13047-014-0037-6

**Published:** 2014-08-22

**Authors:** Carmen-Belén Martínez-Cepa, Juan-Carlos Zuil-Escobar, Raquel Chillón-Martínez, José-Jesús Jiménez-Rejano, Inmaculada-Concepción Palomo-Toucedo

**Affiliations:** 1 Department of Nursing and Physical Therapy, Faculty of Medicine, CEU San Pablo University, Avda, Montepríncipe s/n. Boadilla de Monte, Madrid, 28668, Spain; 2 Department of Physical Therapy, Faculty of Physical Therapy, Nursing and Podiatry, Seville University, Avda, Sanchez Pizjuán s/n., Seville, 41009, Spain

**Keywords:** Reproducibility of results, Anthropometry, Foot

## Abstract

**Background:**

Measurement of first and second metatarsal and toe protrusion is frequently used to explain foot problems using x-rays, osteological measurements or palpation-based tests. Length differences could be related to the appearance of problems in the foot. A test-retest design was conducted in order to establish the intra-rater reliability of three palpation-based tests.

**Methods:**

202 feet of physical therapy students and teachers of the CEU San Pablo University of Madrid, 39 men and 62 women, were measured using three different tests. Data were analysed using SPSS version 15.0. Mean, SD and 95% CI were calculated for each variable. A normal distribution of quantitative data was assessed using the Kolmogorov-Smirnov test. The test-retest intra-rater reliability was assessed using an Intraclass Correlation Coefficient (ICC). The Standard Error Mean (SEM) and the Minimal Detectable Change (MDC) were also obtained.

**Results:**

All the ICC values showed a high degree of reliability (Test 1 = 0.97, Test 2 = 0.86 and Test 3 = 0.88) as did the SEM (Test 1 = 0.07, Test 2 = 0.10 and Test 3 = 0.11) and the MDC (Test 1 = 0.21, Test 2 = 0.30 and Test 3 = 0.31).

**Conclusions:**

Reliability of measuring first and second metatarsal and toe protrusion using the three palpation-based tests showed a high degree of reliability.

## Background

Measurement of the relative difference between the lengths of the first and second toes as well as the first and second metatarsals is commonly used to examine the relationship between protrusion differences and foot problems such as metatarsal stress fractures [1], myofascial trigger point (MTP) activation [2], hallux rigidus [3], hallux valgus [4], hyperkeratosis [5] and midfoot arthrosis [6].

Several studies have utilised x-ray methodology [4],[7]-[11] in order to estimate relative metatarsal length. However, this method has many disadvantages due to cost, accessibility and ionising radiation exposure [12],[13]. Osteological methods are based on direct bone measurements [14]-[16] on cadaveric models, which could be influenced by the presence of necrosis [17]. Finally, clinical palpation is frequently used to identify metatarsal head position. However, many studies do not establish or describe the reliability and validity of this method. Spooner *et al.*[13] used clinical palpation in comparison to radiological measurements in order to establish metatarsal formula. Glasoe *et al.*[18] measured the relative length of the first and second metatarsals, using a caliper and reference bone marks such as the navicular tubercle and the dorsal crease of the first and second metatarsophalangeal joints, observing a poor inter-rater reliability. Based on this method, Davidson *et al.*[12] used a caliper in order to assess first and second toe and metatarsal length differences. They measured 36 feet of 18 participants, performing three different tests, one of which is described by Glasoe *et al.*[18]*.* The aim of this research is to use a larger sample to study the intra-rater reliability of these three methods used by Davidson *et al.*[12]*.*

## Methods

### Procedure

The study included 202 feet (101 right feet and 101 left feet) of 101 (39 men and 62 women) physical therapy students and teachers of the CEU San Pablo University of Madrid. Participants volunteered in response to a poster campaign and consecutive sampling was used. At the beginning of the first session each participant was informed of the aims of and procedure for the study, and completed a consent form before being included. This project was approved by the Research Ethics Committee of the CEU San Pablo University of Madrid. A clinical examination was made by one of the researchers and each participant had to answer a questionnaire in order to know if they fulfilled the inclusion criteria, which were: age between 18 and 40 years, no deformities of the forefoot, no lower limb deformities or congenital illnesses, no degenerative osteoarticular diseases or muscular imbalance, absence of foot pain, no previous foot surgery, no trauma to the foot within the previous 12 months and no signs of alteration of forefoot load distribution. A test-retest design was conducted, so the primary investigator (CBM) performed the three measurement tests on both feet during the first session and repeated the whole procedure 24–48 hours later. All measurements were performed by a physical therapist with fifteen years of experience. All data were recorded using different sheets of graph paper to avoid subjectivity [19].

**Figure 1:**
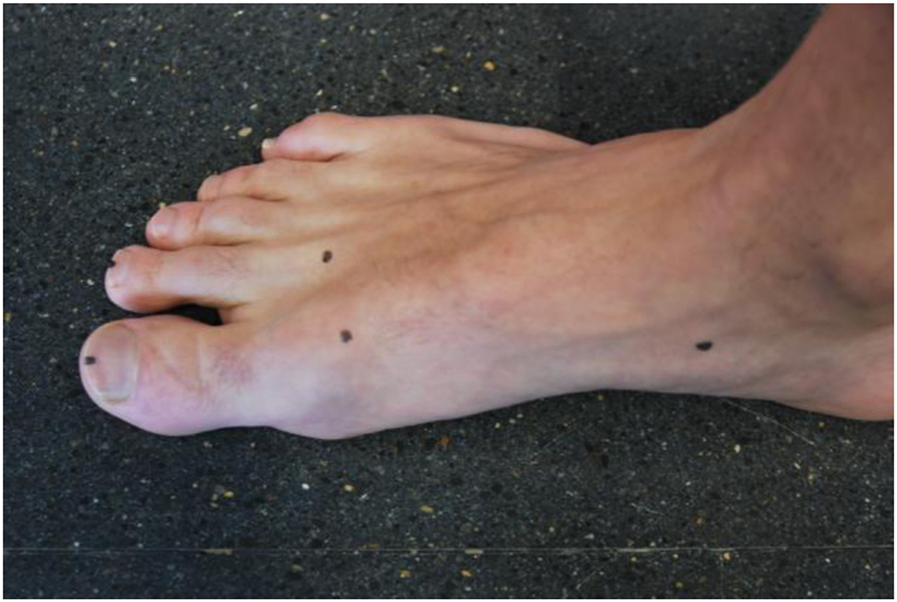
Standardised foot markings.

Using the method described by Davidson *et al*. [12], the dorsal crease of the first and second metatarsophalangeal joints as well as the medial aspect of the navicular tuberosity were identified and marks made at each of these bony landmarks (Figure 1). A vertical reference line, between one point at the base of the calcaneum and 5 cm above this point, was drawn to bisect the posterior calcaneum. Two lines were also drawn down the middle of the first and second toenail of each foot.

**Figure 2:**
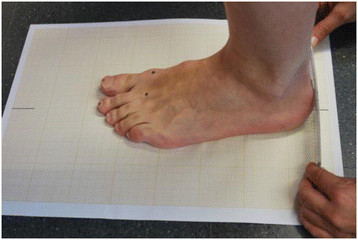
Standard foot position.

All subjects stood on a piece of graph paper with their heels against the edge of a ruler that was aligned with a single horizontal line on the graph paper. The foot was positioned so that the marks on the second toe and calcaneum were in a straight line on the graph paper [19] (Figure 2).

### Test 1: measurement of differences between the end of the first and second toes

**Figure 3:**
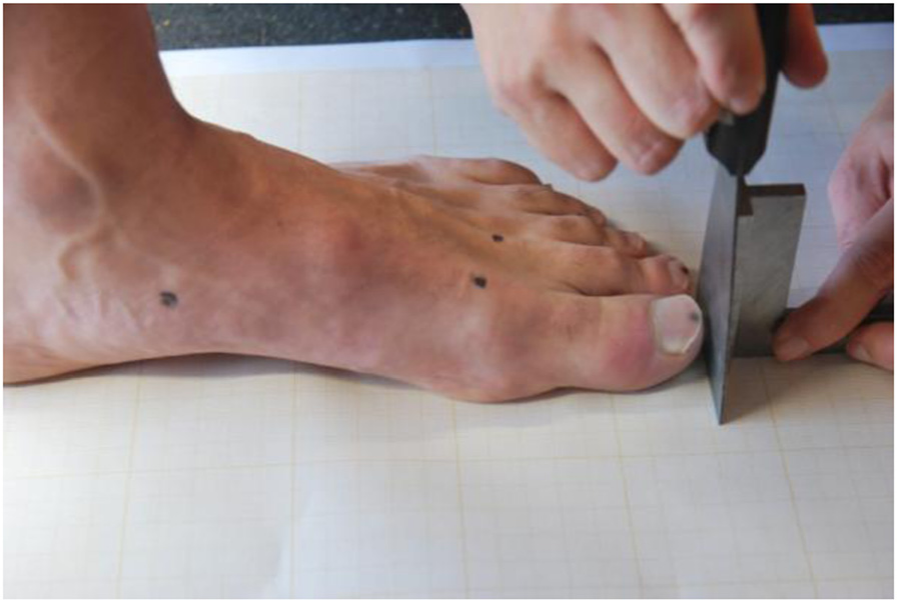
Marking the end of first and second toes.

**Figure 4:**
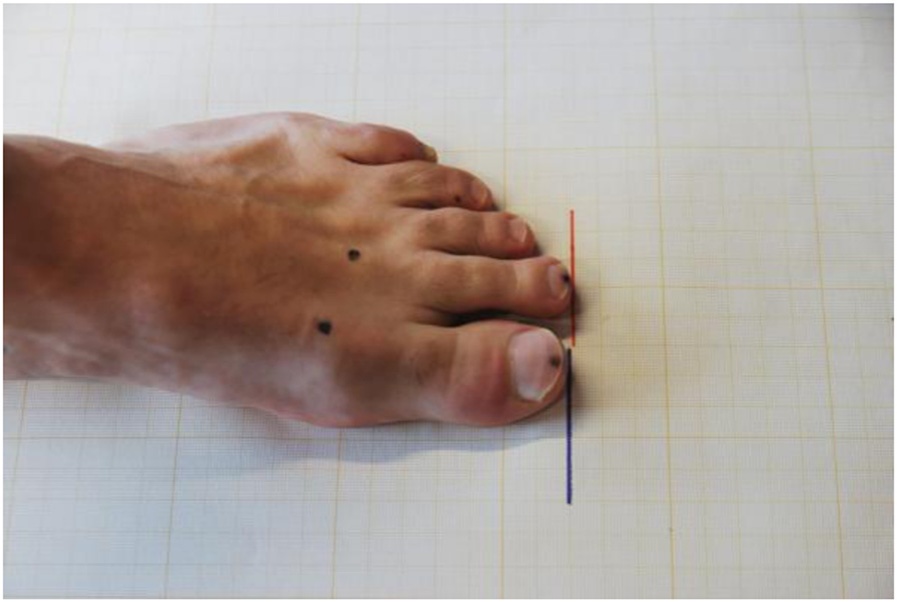
**Lines at the end of first and second toes.** A blue line was made for the first toe and a red line for the second one because in this foot the protrusion is very similar.

Once the foot was placed in the reference position, a carpenter’s square was placed at the end of the first toe in order to keep it aligned with the lines of the graph paper. The investigator then used a paint spatula that had been dampened using a blue ink stamp pad, to make a blue line parallel to the graph lines at the end of the toe, between the first toe and the edge of the square. The procedure was repeated under identical conditions with the second toe (Figure 3). The difference between the ends of both toes was determined using a ruler in order to measure in millimeters the distance between the two lines [12] (Figure 4). If the difference was greater than zero the first toe was longer (Egyptian foot), if it was equal to zero both toes were of identical length (square foot), and if the result was less than zero, the second toe was the longest (Greek foot). Differences of between ±2 mm in this first test were considered to correspond to square foot (first and second toe of identical length) [3],[19],[20]. This test showed excellent intra-rater reliability in the investigation developed by Davidson *et al.*[12].

### Test 2: measurement of differences between the end of the first and second metatarsals

**Figure 5:**
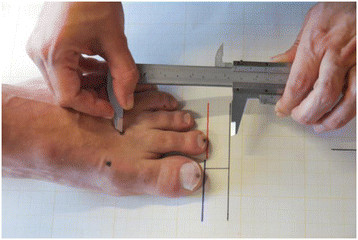
Test 2.

With the foot in the reference position, a black horizontal line was drawn parallel to, and 2 cm away from, the initial line made at the end of the first toe. A sliding vernier caliper was used to measure the distance between the black line and the dorsal crease of the first metatarsophalangeal joint (distance A). The procedure was then repeated, measuring the distance from the end of the second toe to the dorsal crease of the second metatarsal (distance B) (Figure 5). The difference between the lengths of the two metatarsals was then obtained by subtracting distance A from distance B [12]. If the difference was greater than zero the first metatarsal was longer (index plus foot), if it was equal to zero both metatarsals were of identical length (index plus minus foot), and if the result was lower than zero, the second metatarsal was the longest (index minus foot). Differences of between ±2 mm in this test were considered to correspond to an index plus minus foot (first and second metatarsal of identical length) [12],[20].

### Test 3: measurement of differences between the end of the first and second metatarsals and the navicular tubercle

This test has been described by Glasoe *et al.*[18]*.* With the foot in the reference position, the distance between the medial navicular tuberosity marking and the dorsal crease of the first metatarsophalangeal joint was measured using a sliding vernier caliper. This was then repeated, measuring the distance between the medial navicular tuberosity marking and the dorsal crease of the second metatarsal. The relative length difference, in millimeters, is obtained by subtracting the length of the first metatarsal from the length of the second metatarsal [12].

### Statistical analysis

Data were collected and analysed with SPSS 15.0. Each foot was considered as a separate foot. All data was tested for normality using the Kolmogorov-Smirnov test considering normal values of p > 0.05.

The strength of the test-retest intra-rater reliability was assessed using an Intraclass Correlation Coefficient (ICC). The ICC was carried out under a mixed effect and absolute agreement consistency 2 factor alpha model. Using confidence intervals (CI) of 95% for the group mean, an ICC was calculated based on the difference between the means of paired scores for each of the three tests. The standard error mean (SEM) [21] and the minimal detectable change (MDC) were also obtained.

## Results

The sample consisted of 39 men and 62 women with a mean age of 23.7 (5.6) years, a mean body weight of 69.3 (15.5) kg, a mean height of 1.69 (0.96) m and a mean BMI of 24.0 (4.7) kg/m^2^. The intra-rater reliability of the three different tests (ICC, SEM [20] and MDC) is shown in Table 1. For the three tests, the ICCs are significant (p < 0.001). All the ICC values show a high degree of reliability [22]. The small SEM and MDC values revealed a high level of accuracy. The value of the difference in millimeters as well as the percentage of accuracy, up to ±1 mm, obtained in the three tests is shown in Table 2.

**Table 1:** Intra-rater reliability of the three tests

	**ICC**	**CI 95%**	***P*-values**	**SEM**	**MDC**
TEST-RETEST 1	0.97	0.97 to 0.99	<0.001	0.07	0.21
TEST-RETEST 2	0.86	0.80 to 0.90	<0.001	0.10	0.30
TEST-RETEST 3	0.88	0.82 to 0.92	<0.001	0.11	0.31

**Table 2:** Difference between and percentage accuracy of the three tests

	**Difference in mm**	**Percentage accuracy up to ±1 mm**
TEST-RETEST 1	± 3 mm	85%
TEST-RETEST 2	± 5 mm	62%
TEST-RETEST 3	± 5 mm	64%

The different means of toe and metatarsal protrusion are shown in Table 3.

**Table 3:** Toe and metatarsal protrusion means of the three tests

	**First versus second**	**Second versus first**
TEST-RETEST 1	1.30 mm	1.35 mm
TEST-RETEST 2	1.75 mm	1.45 mm
TEST-RETEST 3	1.60 mm	1.60 mm

## Discussion

The aim of this research was to determine the intra-rater reliability of these three tests used to measure toe and metatarsal protrusion of the feet.

The first test, measuring the difference between the ends of the first two toes, showed the highest reliability rate (ICC = 0.97), which is similar to the results obtained by Davidson *et al.* (ICC = 0.98) [12], although the sample used in this study is larger (202 feet versus 36 feet). Using this first palpation-based test, the mean protrusion of the first toe versus the second (Egyptian foot) and the second toe versus the first (Greek foot), was 1.30 and 1.35 mm, respectively. The ±3 mm difference in the results of this first test and the 85% accuracy up to 1 mm demonstrate the precision of this technique.

For the second test, Spooner et *al.*[13] marked the metatarsal heads when standing, in order to compare the results with radiographs. No statistical differences were found between the two methods of measuring length differences, but they did find errors in participants with the two first metatarsals of equal length. The ICC of 0.87 for this second test in our study showed a higher degree of reliability than that obtained by Davidson *et al.* (ICC = 0.76) [12]. The difference of ±5 mm in the results of this second test and the 62% accuracy up to 1 mm show that this technique is less specific than the first test. However, the differences of ±5 mm are smaller than those found by Davidson *et al*. [12] of up to 12 mm. Despite the accuracy of the technique, the palpation of the metatarsal heads and the caliper position may alter the results [12],[19]. Investigators should perform the test in an identical position in order to avoid measurement errors. For this reason, this is the most difficult technique for determining intra-rater reliability. In this second palpation-based test, the mean protrusion of the first metatarsal versus the second (index plus foot) and the second metatarsal versus the first (index minus foot), was 1.75 and 1.45 mm, respectively. In their study of 7167 feet using the radiographic method in 1949, Harris and Beath [10] found a first-second and second-first metatarsal protrusion of 3 mm, with differences of between 1 and 12 mm. Hardy and Clapham [9], with a control group of 504 feet in 1951, and Mancuso *et al.*[23], with a group of 100 healthy feet in 2003, found a first-second metatarsal mean of 2 mm**.** In 2006, Domínguez *et al.*[7] found a second-first metatarsal mean of 1.88 mm. However, despite producing very similar results, the methods are different and not comparable.

For the third test, the ICC of 0.88 is higher than that obtained by Davidson *et al.*[12]. The difference of ±5 mm in the results of this second test and the 64% accuracy up to 1 mm shows that this technique is less specific than the first test. However, the differences of ±5 mm are less than the differences found by Davidson *et al.*[12] of up to 10 mm. The radiological technique used by Hardy and Clapham [9], Mancuso *et al.*[13] and Domínguez *et al.*[7], is the most similar to that used in our study. This test may also be affected by the palpatory identification and the marking of the bony landmarks (metatarsal heads and navicular tubercle) [12],[19]. This test was first performed by Glasoe *et al.*[18], who found a poor inter-rater reliability (ICC = 0.36) in a sample of 15 subjects.

One of the strengths of this study was the confirmation of the greater intra-rater reliability of these three manual tests in determining toe and metatarsal protrusion first demonstrated by Davidson *et al.*[12] using only 36 feet. These tests could be useful in clinical practice, especially when clinicians are dealing with patients involved in physical activities. For example, a first longer toe may cause hallux valgus [4] and is the best foot for ballet dancers [3],[5] with less pain and corns, while a second toe equal or longer than the first toe, is related to pain and swelling over the first metatarsophalangeal joint [3],[5] and hallux rigidus [3]. In contrast, a correctly structured foot is the one in which both metatarsal have the same length or the first is longer than the second [24]. An index minus foot could be related to Morton’s foot structure [2],[10], hallux valgus [24], Freiberg’s disease [25] and second metatarsal stress fractures in ballet dancers [26],[27]. In marathon runners, an index minus foot could be related to activation of peroneus longus myofascial trigger points (MTrPs) due to abnormal weight distribution and excessive pronation of the foot [12],[28],[29]. Other muscles of the lower limb, whose muscles’ MTrPs could be activated are the vastus lateralis, tensor fasciae latae, gemellus superior and inferior, soleus, tibialis anterior and the peroneus brevis [2],[28]. However, an excessively index plus foot has been associated with hallux rigidus [26], hallux limitus [30] and hallux valgus [4],[7],[9],[20],[23],[31].

This study needs to be considered in light of three limitations. One limitation in this study was the inability to compare these tests with osteological or radiological methods in search of the gold standard. Our goal in the near future is to carry out further research in order to determine its validity and to study the inter-rater reliability of the three tests, previously studied by Glasoe *et al.*[18] for the third test. A second limitation is related to the sample of the study consisting of young, healthy adults aged 20–40 years instead of a younger population with growth physes still open, or older or diseased populations with deformities of the forefoot that could affect the results of the study. The final limitation to mention is the approach of analysing 202 feet from 101 participants as separate data items. This assumes that the characteristics of one foot are independent from the characteristics of the other foot, but while it is more common to have the same digital (76.2% versus 23.8%) and metatarsal formula (75.3% versus 24.7%) in both feet, sometimes there are differences. For example, a right Egyptian index-minus foot and a left square index-minus foot in the same subject [19]. To overcome this issue, often only one foot per participant should be selected for inclusion in the analyses [32].

## Conclusions

Reliability of measuring first and second metatarsal and toe protrusion using the three palpation-based tests showed a high degree of reliability for all the ICC values. Being simple, cheap and non-invasive, palpation-based methods can be used by clinicians to measure metatarsal and toe protrusion in clinical practice.

## Competing interests

The authors declare that they have no competing interests.

## Authors’ contributions

CBM conceived of the study, was responsible for the design and data acquisition, contributed to the analyses and drafted the original manuscript. JZ contributed to the design, analysis and interpretation of data and the revision of the manuscript. JJ carried out the statistical analyses, contributed to the interpretation of data and the revision of the manuscript. RC and IP made substantial contributions to the drafting and revision of the manuscript. All authors read and approved the final version of the manuscript.
